# Medical College Notes

**Published:** 1889-07

**Authors:** 


### Medical College Notes.

University of Buffalo.—Dr. Charles Cary, Professor of Anatomy, has been appointed Professor of Materia Medica and Therapeutics, *vice* E. V. Stoddard, resigned.

Niagara University.—The following changes are announced: Dr. John A. Miller, Lecturer on Chemistry, has been appointed Professor of Medical Chemistry and Toxicology; Dr. C. C. Frederick,

Assistant to Chair of Obstetrics, becomes Adjunct Professor of Obstetrics; Dr. F. A. Harrington, Assistant in Materia Medica, is chosen Lecturer on Hygiene; Dr. A. Melville Ewing is appointed Lecturer on Pathology and Dermatology; and Dr. Henry C. Buswell is elected Assistant in Materia Medica.

